# Ursodeoxycholic Acid Improves Bilirubin but Not Albumin in Primary Biliary Cirrhosis: Further Evidence for Nonefficacy

**DOI:** 10.1155/2013/139763

**Published:** 2013-07-24

**Authors:** Emmanuel A. Tsochatzis, Maurille Feudjo, Cristina Rigamonti, Jiannis Vlachogiannakos, James R. Carpenter, Andrew K. Burroughs

**Affiliations:** ^1^The Royal Free Sheila Sherlock Liver Centre, Royal Free Hospital and UCL Institute of Liver and Digestive Health, London NW3 2QG, UK; ^2^UK Medical Statistics Unit, London School of Hygiene and Tropical Medicine, Keppel Street, London WC1E 7HT, UK

## Abstract

*Background/Aim.* In randomised controlled trials (RCTs) of ursodeoxycholic acid (UDCA), although serum bilirubin is frequently reduced, its effect on disease progression and mortality is unclear. As serum albumin is an established independent prognostic marker, one might expect less deterioration of serum albumin values in a UDCA-treated group. We therefore modelled the typical evolution of serum bilirubin and albumin levels over time in UDCA-untreated patients and compared it with the observed levels in UDCA RCTs. *Methods*. Multilevel modelling was used to relate the evolution of serum albumin to serum bilirubin and time since patient referral. For each considered RCT, the derived model was used to predict the relationship between final mean serum albumin and bilirubin concentration, adjusted for mean serum albumin at referral and followup duration. *Results*. Five RCTs were eligible in terms of available data, of which two had long followup. In all trials, serum albumin did not significantly differ between UDCA- and placebo-treated patients, despite the UDCA effect on serum bilirubin. Therefore, there is no evidence over time for changes or maintenance of albumin levels for UDCA-treated patients above the levels predicted for placebo-treated patients. *Conclusions*. Our findings suggest that UDCA does not alter serum albumin in a way that is consistent with its effect on serum bilirubin. Therefore, reductions in serum bilirubin of UDCA-treated PBC do not parallel another validated and independent prognostic marker, further questioning the validity of serum bilirubin reduction with UDCA as a surrogate therapeutic marker.

## 1. Introduction

Primary biliary cirrhosis (PBC) is a slowly progressive disease characterised by the destruction of smaller size bile ducts leading to hepatic fibrosis, cirrhosis, and eventually liver failure [1]. Currently, the only approved treatment is ursodeoxycholic acid (UDCA), which is claimed to modify disease progression [2]. However, separate meta-analyses have not shown benefit of UDCA in terms of survival, transplantation rates, or complications of disease [3–5].

 Serum bilirubin and albumin levels are well-established and important prognostic markers for time to death or transplantation in untreated patients, being universally present in all disease prognostic models [6–8]. However, in the presence of treatment, an objective assessment of their quality and reliability as surrogate endpoints for survival has not been carried out despite adequate published methodology [9–11]. Although a serum bilirubin rise is clearly linked to shortened survival, the effect of a UDCA-mediated decrease is unclear [3–5], as patient survival and disease progression are not demonstrably improved. Therefore, it can be considered premature and possibly unreliable to draw conclusions on the efficacy of treatment based solely on serum bilirubin. This would be particularly pertinent when considering UDCA, as other surrogate endpoints have not been shown to be different between UDCA and placebo or no treatment in randomised trials. 

In order to further assess the benefit of UDCA as a specific therapy in PBC, we considered the relationship between serum bilirubin and albumin over time in a cohort of untreated patients, with the expected rise in bilirubin being associated with a fall in serum albumin. We then evaluated this relationship in the UDCA trials with the expectation of observing a slower fall in serum albumin in the UDCA groups, if indeed treatment was delaying disease progression. Absence of this effect would raise further doubt on the true impact of UDCA as a disease modifier in PBC, particularly if this were to be true in trials with very long followup and substantial reductions in serum bilirubin concentrations in the UDCA-treated groups.

## 2. Patients and Methods

We used a cohort of PBC patients untreated with UDCA and independent of randomised trials in order to describe the evolution of serum bilirubin and albumin levels over time. This reference cohort consisted of 289 patients referred to the Royal Free Hospital, London, between January 1, 1977, and December 31, 1989. Full details are given elsewhere [17, 18]. All patients were untreated with UDCA and presented without variceal bleeding, a criterion of exclusion in the randomised trials of UDCA. Patients were followed up from referral for a median of 3.3 years (10th and 90th percentiles of 0.2 and 9.1 years). During followup, serum bilirubin and albumin levels were routinely monitored, usually every 3–6 months. The average number of measurements per patient was 18 (10th and 90th percentiles of 6 and 33). The means (standard error) of bilirubin and albumin concentrations at referral were 33.7 umol/L (4.87) and 39.9 g/L (0.42), respectively. 

Using the reference cohort, we modelled the repeated measurements of serum albumin and log_10_ (bilirubin), together with time from referral, to establish the typical relationship between them. We investigated whether this relationship depended on age and sex. A random effects model was used [8], which is a classical model for repeated measures. For each subject, all successive measurements of bilirubin and albumin together with the time of measurement are considered. A hierarchical modelling approach was adopted to allow subject specific profiles to be modelled. It is clear that this guarantees not only individual predictions but also the prediction of the means. Model building was carried out using backward selection, with testing at the 5% level of significance, to arrive at the simplest satisfactory model for the mean and variance structure. Despite considering the simplest structure for the residuals variance, our model with both random intercept and random slope on time ensures that correlation between repeated measurements is appropriately accounted for. The statistical software MLwiN version 2.1d was used [19].

It might be considered that dropouts might affect our results and interpretation of the relationship between serum bilirubin and albumin concentrations, particularly if a dropout could not be predicted using earlier response values or variables. Our modelling adopted a likelihood approach, which enables an unbiased estimate even when the patients dropping out may be those with a particular bilirubin or albumin profile. Indeed, the modelling approach does not assume that dropouts are occurring completely at random (i.e., dropouts not related to disease progression) but allows for this, eliminating potential bias. 

Having established the typical relationship between albumin, log_10_ (bilirubin), and time from referral in patients untreated with UDCA, we compared this relationship with that seen in clinical trials of UDCA included in the meta-analysis [5] and from UDCA trial reports [12–16]. This was done as follows. For each eligible trial, our reference cohort model was used to predict the albumin levels one would expect to see at the end of the trial, given the initial albumin, final bilirubin, and length of followup. For this purpose, “time since randomisation” in the clinical trial was equated to “time since referral” in the reference cohort. We calculated 95% confidence intervals for our albumin concentration predictions and compared them with the published 95% confidence intervals for the end of trial albumin concentrations. We only included UDCA trials in which details of biochemical values were available at the start and end of the therapy. 

## 3. Results

### 3.1. Model for Reference Cohort

The final model related serum albumin *t* days after referral to (i) bilirubin *t* days after referral, (ii) *t* the number of days after referral, and (iii) albumin at referral. Differences in this relationship by sex and age were not statistically significant. The model is as follows:

(1) albumin  t  days  after  randomisation  (g/L)=β0 +β1×log⁡10⁡(bilirubin  t  days  after  referral  (μmol/L)) +β2×{log⁡10⁡(bilirubin  t  days  after  referral  (μmol/L))}2 +β3×t+β4x(albumin  at  baseline  (g/L)),

with fixed parameter estimates shown in Table 1, random parameter estimates shown in the Appendix.

### 3.2. Evaluation of UDCA Trials

Six of the 11 identified UDCA trials in the meta-analysis [5] could not be evaluated as they gave no information on serum albumin levels at randomisation nor at the end of followup [20–25]. Furthermore, although some of the 5 eligible trials had additional longer followup [12–16], with use of UDCA (placebo patients changed to UDCA), unfortunately, there was no information on either serum albumin or serum bilirubin levels at the end of the conversion phase on UDCA therapy, so this additional followup could not be used. 

As previously described, we identified 5 eligible trials (Table 2). Two trials had long followup (Papatheodoridis et al. [16], median followup 7 years, and Pares et al. [15], median followup 3.4 years). Three trials had short followup (Vuoristo et al. [14], median followup 2 years; Poupon et al. [12], median followup 2 years; and Battezzati et al. [13], median followup 9 months). Firstly we focused on the two trials with the longer followup. 

**Figure 1:**
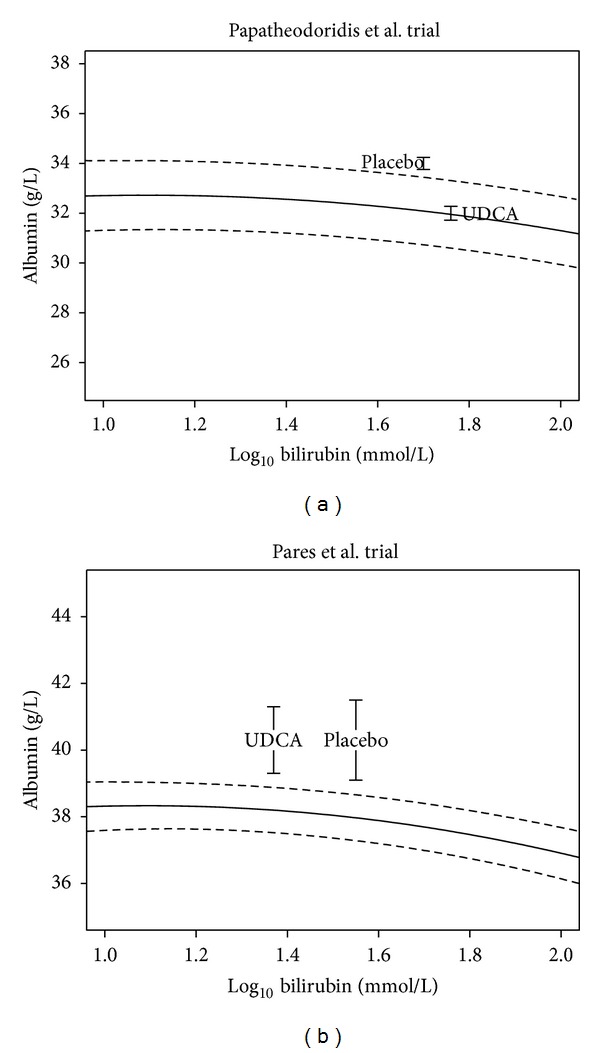
Reported and predicted levels of serum bilirubin and serum albumin for trials with long follow-up period. Vertical lines represent 95% confidence intervals. First (a) shows the profile of the relationship between serum bilirubin and albumin (solid line) with the 95% confidence interval (dash line) predicted by the model applied to the trial reported by Papatheodoridis et al. [16]. This trial had a baseline albumin of 36 g/L and 37 g/L for UDCA and untreated arms, respectively, and a mean followup of 7 years. As the number of patients who were initially assigned to remain untreated were crossed over to UDCA therapy at some point during the study period, the end of followup was considered the time of crossover for those patients. Superimposed on this in the figure is a point corresponding to the end of trial albumin and bilirubin level for each of the trial arms (UDCA and placebo), together with the reported 95% confidence interval for albumin. Likewise, (b) shows the corresponding results for the trial reported by Pares et al. [15], which had a baseline serum albumin of 41.9 g/L and 40.6 g/L for UDCA and placebo arms, respectively.

In the trial by Papatheodoridis et al. [16] (Figure 1(a)), the model from the independent reference cohort for UDCA untreated patients closely predicts the end of trial albumin concentration in those treated with UDCA. In other words, UDCA did not improve the albumin level of patients over and above that seen in the untreated reference cohort with a similar followup, baseline albumin, and final bilirubin. Conversely, the placebo arm shows a significant improvement in albumin against the independent reference cohort. This difference in serum albumin concentrations at the end of the followup between UDCA, and placebo-treated groups with respect to the independent reference cohort is consistent with UDCA having no effect on albumin, a known prognostic marker in PBC.

In the UDCA arm in the Pares et al. trial [15] (Figure 1(b)), the serum albumin does improve over and above that predicted by the reference cohort. However, the placebo arm has a more marked improvement in albumin, being further above the predicted curve. Therefore, although UDCA has reduced serum bilirubin, there is no corresponding improvement in serum albumin over and above the level seen in the placebo arm. This is also consistent with no UDCA effect on underlying prognosis, as measured by albumin levels.

**Figure 2:**
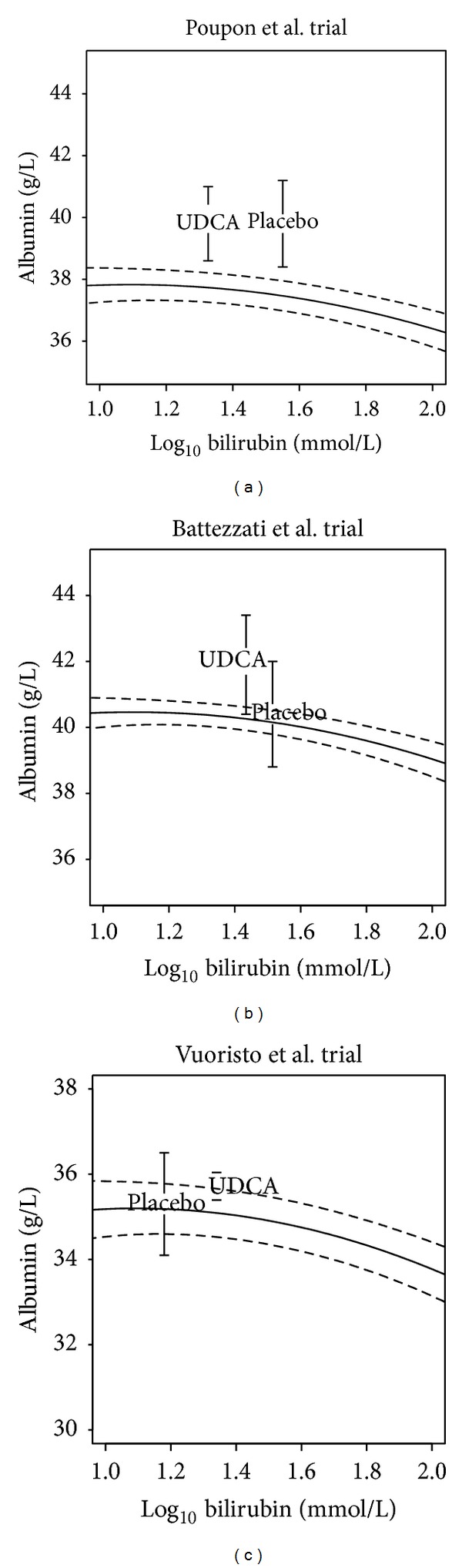
Reported and predicted levels of serum bilirubin and serum albumin for trials with short follow up period. Vertical lines represent 95% confidence intervals. In the Battezzati et al. [13] (b) and Vuoristo et al. [14] (c) trials, when compared with the placebo-treated arm, the UDCA-treated patients have a serum albumin concentration further above the expected value suggested by their bilirubin. However, in both these studies the difference between the predicted and observed results does not approach statistical significance at the 5% level. By contrast, the study by Poupon et al. [12] (a) shows that the placebo-treated patients have a mean albumin level further above their predicted level than the UDCA patients.

The results for the three trials with shorter followup are shown in Figure 2. In the Vuoristo et al. [14] and the Battezzati et al. [13] trials, when compared with the placebo-treated arm, the UDCA-treated patients have a serum albumin concentration further above the expected value suggested by their bilirubin. However, in both of these studies, the difference between the predicted and observed results does not approach statistical significance at the 5% level. By contrast, the study by Poupon et al. [12] shows that the placebo-treated patients have a mean albumin level further above their predicted level compared to UDCA patients. Again, this is consistent with no effect of UDCA on underlying prognosis, as measured by albumin levels.

## 4. Discussion

In the present study, we assessed the statistical relationship between serum bilirubin and albumin concentrations during the natural course of PBC in untreated patients and further constructed a time model on their relation, since both are well-established independent predictors of survival and thus disease progression in PBC. We then compared the relationship seen in the reference cohort between albumin, bilirubin, and time since referral with that seen in published UDCA trials. Specifically, the reported reduction in serum bilirubin levels in UDCA trials was assessed in relation to an accompanying and corresponding slower reduction, stabilisation or even increase in serum albumin concentration, consistent with improved prognosis. 

As a higher concentration in serum albumin is associated with improved survival, and if UDCA not only lowers bilirubin, but also improves prognosis, one would expect to see a slower fall in the level of albumin (after adjusting for time and baseline albumin). Therefore, if the observed albumin at the end of the trial's followup was lower than that predicted by the model, given the final bilirubin level and the length of followup, this would be consistent with UDCA having no effect on prognosis. Conversely, if it were higher than that predicted by the model, this would be consistent with UDCA improving prognosis.

Our model was thus used to predict the serum albumin concentrations at the conclusion of the five randomised trials of UDCA that reported end of study albumin, and it compared these predictions with what was actually observed. In all assessed trials, serum albumin did not significantly differ between UDCA- and placebo-treated patients, despite the significant UDCA effect on serum bilirubin. Only three of these trials showed a significantly different bilirubin/albumin relationship to the reference cohort. The trials reported by Poupon et al. [12] and Pares et al. [15] showed no difference in final albumin levels between the trial arms of placebo and UDCA. However, relative to the reference cohort, given the final bilirubin, the final albumin was further above the prediction in the placebo arms for both trials. This is consistent with UDCA not modifying disease progression as measured by albumin concentrations. 

This conclusion is reinforced by the trial by Papatheodoridis et al. [16] which has the longest followup similar to our reference cohort. Here, in the UDCA arm, the final albumin level was closely predicted by our model, whereas in the untreated arm, the albumin level was significantly above that predicted by the model. In the two other studies [13, 14], although the final serum albumin was higher in the UDCA arm compared to placebo, the difference between the predicted and observed results did not reach statistical significance. 

Therefore, the results of these 5 studies show that UDCA does not affect the disease progression as measured by serum albumin, and this further supports the results and interpretation published meta-analysis [3–5] which fail to demonstrate a beneficial effect of UDCA.

Our findings clearly suggest that serum bilirubin cannot be taken as a validated surrogate marker of therapeutic benefit. It is recognised that bilirubin concentrations fall within 3 months in UDCA-treated patients and then rise less rapidly than in nontreated patients. This response can be attributed to the choleretic effect of UDCA, that is, stimulation of biliary secretion of bile acids [26], and appears to be merely biochemical, as it does not result in an improvement in liver synthetic function or in patients' survival.

The limitations of our approach could be due to possible differences between the independent reference cohort and the study populations. However, although we would expect a cohort referred to a hospital to be more severely ill than patients taking part in trials, we did exclude patients with bleeding as a presentation as did the trials. The mean bilirubin and albumin concentrations in our cohort at referral were almost the same as those reported by Battezzati et al. [13]. The bilirubin and albumin relationship was closely predicted by our model in the trial with the longest followup [16], although bilirubin values were lower. Thus, it is reasonable to expect the pattern of the relationship to be similar in the various groups of patients, thus making our interpretation valid. Another potential problem is the change in the assay techniques for serum albumin over times, which are not detailed in the reports. Although this could affect the comparison with respect to the reference cohort, the comparison within a single trial of UDCA and placebo/untreated groups with respect to the reference cohort is still valid and not biased by changes in measurement methodology. This may be the reason for higher than predicted albumin values in the placebo/untreated groups.

We did not evaluate the relationship of serum bilirubin concentrations with the other known or reported prognostic factors in PBC; the data on prothrombin time was not available in the randomised trials of UDCA, and neither in our database, as prothrombin time values are not routinely measured during followup of PBC patients. Histological progression, although considered by some to be affected favourably by UDCA but not by us [27], cannot easily be assessed, as usually no more than one biopsy is available in routine clinical practice, and by no means all patients in the UDCA trials had paired biopsies. Lastly, as regards oedema and/or ascites, it is difficult to model this variable, as assessing progression of this is difficult to measure, as compared with the continuous variables of serum bilirubin and albumin. 

We conclude that there is no evidence that UDCA acts on serum albumin concentrations in a way that is consistent with its effect on serum bilirubin levels. Given that these are both independent prognostic markers in PBC and that lower serum bilirubin concentrations are associated with higher serum albumin concentrations, this result supports the evidence and the interpretation for the lack of effect of UDCA on PBC disease progression as detailed in independent meta-analyses [3–5]. Therefore, reduction in serum bilirubin concentration in UDCA-treated PBC patients cannot be taken as a validated surrogate marker of therapeutic benefit. 

## Figures and Tables

**Table 1:** Model parameter estimates.

Parameters	Estimate (standard error)	Description
β_0_	9.57 (1.63)	Intercept
β_1_	3.73 (1.15)	log_10_ (bilirubin *t* days after referral (*μ*mol/L))
β_2_	−1.68 (0.35)	{log_10_ (bilirubin *t* days after referral (*μ*mol/L))}
β_3_	−2.3 × 10^−3^ (4.85 × 10^−4^)	Time
β_4_	0.720 (0.031)	Albumin at baseline (g/L)

**Table 2:** Mean ± standard error of serum albumin (g/L) and bilirubin (*μ*mol/L) levels at baseline and end of the followup (before crossover, if any) for considered trials.

	First bilirubin UDCA/no UDCA	Last bilirubin UDCA/no UDCA	First albumin UDCA/no UDCA	Last albumin UDCA/no UDCA	Median followup
Poupon et al. [12], 1991	23.2 ± 2.6 *μ*mol/L 21.2 ± 2.5 *μ*mol/L	12.3 ± 0.9 *μ*mol/L 17.9 ± 2.2 *μ*mol/L	38.9 ± 0.5 g/L 40.1 ± 0.5 g/L	39.8 ± 0.6 g/L 39.8 ± 0.7 g/L	24 months

Battezzati et al. [13], 1993	31.5 ± 4.0 *μ*mol/L 32.5 ± 3.7 *μ*mol/L	27.2 ± 3.4 *μ*mol/L 32.8 ± 4.7 *μ*mol/L	40.7 ± 1.0 g/L 40.9 ± 0.8 g/L	41.9 ± 0.9 g/L 40.4 ± 0.8 g/L	9 months

Vuoristo et al. [14], 1995	22.7 ± 3.4 *μ*mol/L 25.5 ± 6.8 *μ*mol/L	20.4 ± 6.8 *μ*mol/L 15.3 ± 1.7 *μ*mol/L	35.0 ± 0.7 g/L 35.6 ± 0.7 g/L	35.7 ± 0.1 g/L 35.3 ± 0.6 g/L	25 months

Pares et al. [15], 2000	22.1 ± 1.7 *μ*mol/L 27.2 ± 3.4 *μ*mol/L	23.8 ± 3.4 *μ*mol/L 35.7 ± 3.1 *μ*mol/L	41.9 ± 0.6 g/L 40.6 ± 0.6 g/L	40.3 ± 0.5 g/L 40.3 ± 0.6 g/L	3.4 years

Papatheodoridis et al. [16], 2002*	23.8 ± 0.78 *μ*mol/L 22.1 ± 1.02 *μ*mol/L	35.7 ± 5.7 *μ*mol/L 39.1 ± 8.5 *μ*mol/L	37 ± 5 g/L 38 ± 4 g/L	32 ± 6 g/L 34 ± 5 g/L	7 years

Bilirubin values in mg/dL were converted for the statistical evaluation (1 mg/dL = 17.1 *μ*mol/L).

*In this trial, the untreated control group did not receive placebo.

## Appendix

To account for the correlation in the repeated measurements in the data set of the independent reference cohort, the coefficients of log_10_ (bilirubin), time since referral, and the intercept were allowed to be normally distributed about their mean estimated values (shown in Table 1). The estimated variance matrix of this trivariate normal distribution is

(A.1) $$\begin{array}{c} \left(\begin{array}{ccc} 16.93 & 5.01\times 10^{-4} & -13.6\\ 5.01\times 10^{-4} & 7.41 & 9.38\times 10^{-4}\\ -13.6 & 9.38\times 10^{-4} & 30.13 \end{array}\right), \end{array}$$

where the diagonal elements are the variances of log_10_ (bilirubin), time since referral, and the intercept, respectively, and the off-diagonal elements are the covariances between them.

## References

[1] Kaplan MM, Gershwin ME (2005). Primary biliary cirrhosis. *The New England Journal of Medicine*.

[2] Tsochatzis EA, Gurusamy KS, Gluud C, Burroughs AK (2009). Ursodeoxycholic acid and primary biliary cirrhosis: EASL and AASLD guidelines. *Journal of Hepatology*.

[3] Gluud C, Christensen E (2002). Ursodeoxycholic acid for primary biliary cirrhosis. *Cochrane Database of Systematic Reviews*.

[4] Gong Y, Huang ZB, Christensen E, Gluud C (2008). Ursodeoxycholic acid for primary biliary cirrhosis. *Cochrane Database of Systematic Reviews*.

[5] Goulis J, Leandro G, Burroughs AK (1999). Randomised controlled trials of ursodeoxychoric-acid therapy for primary biliary cirrhosis: a meta-analysis. *The Lancet*.

[6] Chan C, Carpenter JR, Rigamonti C, Gunsar F, Burroughs AK (2005). Survival following the development of ascites and/or peripheral oedema in primary biliary cirrhosis: a staged prognostic model. *Scandinavian Journal of Gastroenterology*.

[7] Christensen E, Altman DG, Neuberger J (1993). Updating prognosis in primary biliary cirrhosis using a time-dependent Cox regression model. *Gastroenterology*.

[8] Dickson ER, Grambsch PM, Fleming TR, Fisher LD, Langworthy A (1989). Prognosis in primary biliary cirrhosis: model for decision making. *Hepatology*.

[9] Buyse M, Molenberghs G (1998). Criteria for the validation of surrogate endpoints in randomized experiments. *Biometrics*.

[10] Freedman LS, Graubard BI, Schatzkin A (1992). Statistical validation of intermediate endpoints for chronic diseases. *Statistics in Medicine*.

[11] Prentice RL (1989). Surrogate endpoints in clinical trials: definition and operational criteria. *Statistics in Medicine*.

[12] Poupon RE, Balkau B, Eschwege E, Poupon R (1991). A multicenter, controlled trial of ursodiol for the treatment of primary biliary cirrhosis. *The New England Journal of Medicine*.

[13] Battezzati PM, Podda M, Bianchi FB (1993). Ursodeoxycholic acid for symptomatic primary biliary cirrhosis. Preliminary analysis of a double-blind multicenter trial. *Journal of Hepatology*.

[14] Vuoristo M, Färkkilä M, Karvonen A (1995). A placebo-controlled trial of primary biliary cirrhosis treatment with colchicine and ursodeoxycholic acid. *Gastroenterology*.

[15] Pares A, Caballeria L, Rodes J (2000). Long-term effects of ursodeoxycholic acid in primary biliary cirrhosis: results of a double-blind controlled multicentric trial. UDCA-Cooperative Group from the Spanish Association for the Study of the Liver. *Journal of Hepatology*.

[16] Papatheodoridis GV, Hadziyannis ES, Deutsch M, Hadziyannis SJ (2002). Ursodeoxycholic acid for primary biliary cirrhosis: final results of a 12-year, prospective, randomized, controlled trial. *American Journal of Gastroenterology*.

[17] Hughes MD, Raskino CL, Pocock SJ, Biagini MR, Burroughs AK (1992). Prediction of short-term survival with an application in primary biliary cirrhosis. *Statistics in Medicine*.

[18] Chan C-W, Tsochatzis EA, Carpenter JR, Rigamonti C, Gunsar F, Burroughs AK (2010). Predicting the advent of ascites and other complications in primary biliary cirrhosis: a staged model approach. *Alimentary Pharmacology and Therapeutics*.

[19] Goldstein H, Browne W, Rasbash J (2002). Multilevel modelling of medical data. *Statistics in Medicine*.

[20] Combes B, Carithers RL, Maddrey WC (1995). A randomized, double-blind, placebo-controlled trial of ursodeoxycholic acid in primary biliary cirrhosis. *Hepatology*.

[21] Eriksson LS, Olsson R, Glauman H (1997). Ursodeoxycholic acid treatment in patients with primary biliary cirrhosis. A Swedish multicentre, double-blind, randomized controlled study. *Scandinavian Journal of Gastroenterology*.

[22] Heathcote EJ, Cauch-Dudek K, Walker V (1994). The Canadian multicenter double-blind randomized controlled trial of ursodeoxycholic acid in primary biliary cirrhosis. *Hepatology*.

[23] Leuschner U, Fischer H, Kurtz W (1989). Ursodeoxycholic acid in primary biliary cirrhosis: results of a controlled double-blind trial. *Gastroenterology*.

[24] Lindor KD, Dickson ER, Baldus WP (1994). Ursodeoxycholic acid in the treatment of primary biliary cirrhosis. *Gastroenterology*.

[25] Turner IB, Myszor M, Mitchison HC, Bennett MK, Burt AD, James OFW (1994). A two year controlled trial examining the effectiveness of ursodeoxycholic acid in primary biliary cirrhosis. *Journal of Gastroenterology and Hepatology*.

[26] Ikegami T, Matsuzaki Y (2008). Ursodeoxycholic acid: mechanism of action and novel clinical applications. *Hepatology Research*.

[27] Chan C, Papatheodoridis GV, Goulis J, Burroughs AK (2003). Ursodeoxycholic acid and histological progression in primary biliary cirrhosis. *Journal of Hepatology*.

